# Tako-Tsubo-Kardiomyopathie im Rahmen einer Traumareanimation

**DOI:** 10.1007/s00113-024-01497-z

**Published:** 2024-11-02

**Authors:** Sonja Wassman, Gregor Römmermann, Michael Dommasch

**Affiliations:** 1 Zentrale interdisziplinäre Notaufnahme, Klinikum rechts der Isar, Technische Universität München, Ismaninger Str. 22, 81675 München, Deutschland; 2 Klinik für Unfallchirurgie, Klinikum rechts der Isar, Technische Universität München, Ismaninger Str. 22, 81675 München, Deutschland

## Anamnese

Ein 87-jähriger Patient mit vorbestehender koronarer Zweigefäßerkrankung wird mittels Rettungswagen in Notarztbegleitung intubiert und beatmet nach erfolgreicher kardiopulmonaler Reanimation in unseren Schockraum eingeliefert. Der Patient war als behelmter Motorrollerfahrer mit unbekannter Geschwindigkeit auf einen ausparkenden Kleintransporter aufgefahren. Nach dem konsekutiven Sturz wurde der Patient von den Rettungskräften ohne Vitalzeichen aufgefunden und unmittelbar reanimiert. Die initiale Rhythmuskontrolle zeigte eine pulslose elektrische Aktivität (PEA), welche nach einer Adrenalinbolusgabe von 1 mg nach 10 min im „return of spontaneous circulation“ (ROSC) mündete. Die genaue Ursache des Unfalls ist unklar.

## Befund

Bei Übergabe im Schockraum ist der Patient regelrecht intubiert und seitengleich beatmet, tachykard und hypoton mit konsekutiver Katecholaminpflichtigkeit. Die Pupillen sind eng, isokor und beidseits prompt lichtreagibel. Der Babinski-Reflex ist beidseits negativ. Ein weiterer neurologischer Untersuchungsbefund ist bei analgosediertem und intubiertem bzw. beatmetem Patienten nicht möglich. Es finden sich multiple Schürfwunden und Ablederungsläsionen der Haut im Gesicht und an den Extremitäten.

## Diagnostik

**Abb. 1:**
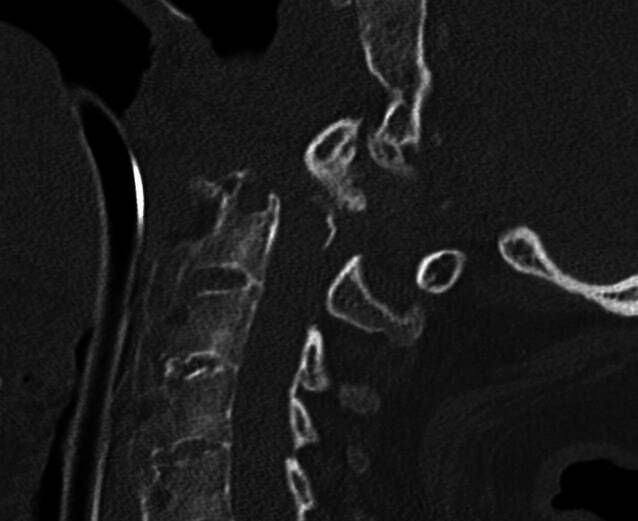
Computertomographie im Schockraum: dislozierte Densfraktur Typ II nach Anderson und D’Alonzo mit atlantoaxialer Luxation

Nach strukturierter Übergabe gemäß Schockraumalgorithmus erfolgt nach 35 min eine Computertomographie (CT), inkl. Cranium und Gesichtsschädel ohne wegweisenden pathologischen Befund. Es zeigt sich im CT der HWS eine dislozierte Densfraktur Typ II nach Anderson und D’Alonzo mit begleitender bilateraler atlantoaxialer Luxation und entsprechender, im MRT bestätigter absoluter spinaler Enge mit Kompression des Myelons auf Höhe von HWK2/3 (Abb. 1). Weiterhin zeigen sich bilaterale Frakturen des Arcus posterius des Atlas, sowie eine DISH-Fraktur auf Höhe HWK7/BWK 1 mit Beteiligung der Deckplatte von BWK 1.

In der Echokardiographie zeigt sich eine hochgradig eingeschränkte linksventrikuläre Ejektionsfraktion (LVEF) mit einem deutlichen „apical ballooning“, das am ehesten im Sinne einer Tako-Tsubo-Kardiomyopathie gedeutet werden kann (s. Zusatzmaterial online: Video). Der rechte Ventrikel sowie die V. cava inferior sind normal konfiguriert. Elektrokardiographisch zeigen sich das Bild eines Linksschenkelblocks sowie ein tachykard übergeleitetes Vorhofflimmern.

Laborchemisch zeigen sich das hs-Troponin T mit 0,019 ng/ml und das Lactat mit 4,8 mmol/l erhöht [1, 4, 7].

## Therapie und Verlauf

Nach initialer Schockraumtherapie und -diagnostik erfolgt die zügige Verlegung in den OP zur Versorgung der dislozierten Densfraktur mittels dorsaler Stabilisierung. Dies erfolgt in komplikationsloser Intubationsnarkose. Der Patient wird mit weiterhin echokardiographisch nachgewiesener, nun global schlechter Pumpfunktion des Herzens sowie Katecholaminpflichtigkeit auf unsere Intensivstation übernommen. Die laborchemische Verlaufskontrolle zeigt weiterhin steigendes hs-Troponin T auf 0,546 ng/ml und Lactat auf 5,1 mmol/l. Hier erfolgt bei fehlender Aufwachreaktion, trotz Sedierungspause, die Anlage einer externen Ventrikeldrainage rechts frontal zur Überwachung des Hirndrucks. Aufgrund der infausten Prognose bei hypoxischem Hirnschaden und hoher Querschnittslähmung mit Verletzung des Hirnstamms entscheiden wir uns interdisziplinär in Rücksprache mit den Angehörigen des Patienten und entsprechend dokumentiertem Patientenwillen für die Einstellung der lebenserhaltenden Therapiemaßnahmen. Der Patient verstirbt 4 Tage später auf unserer Intensivstation.

## Diskussion

Mykokardiale Dysfunktionen können sich bei schwerem Schädel-Hirn-Trauma und Verletzungen des Hirnstamms mit einer unterschätzten Inzidenz zeigen. In unserem Fall zeigt der Patient einen schweren neurogenen Schock als Folge der Hirnstammverletzung bei atlantoaxialer Luxation. Durch die ZNS-Schädigung kann es zur Überstimulation der β‑ und α‑Rezeptoren mit konsekutiver kardialer Dysreflexie und ausgeprägter Hypotension kommen, die in diesem Fall vermutlich die Ursache der Reanimationspflichtigkeit des Patienten war. Auch, wenn die dezidierte Ursache des Unfalls bei unserem Patienten nicht klar ist, liegt eine kardiale Genese dennoch nahe. Zwei Mayo-Clinic-Kriterien für die Tako-Tsubo-Kardiomyopathie sind mit dem echokardiographischen Befund und der milden Erhöhung des Troponins in diesem Fall erfüllt. Aufgrund der infausten Prognose des Patienten wird auf weitere invasive Diagnostik mittels Herzkatheter, welcher unsere Verdachtsdiagnose weiter erhärten hätte können. verzichtet. Auch ist der Beobachtungszeitraum bis zum Versterben des Patienten zu gering, um weitere kardiale Verlaufskontrollen durchführen zu können. Somit bleibt im hier beschriebenen Fall die Tako-Tsubo-Kardiomyopathie trotz eindeutiger echokardiographischer Kriterien nur ein Verdacht. Nichtsdestotrotz ist das Auftreten einer Tako-Tsubo-Kardiomyopathie vorwiegend bei schwerem Schädel-Hirn-Trauma mit intrakranieller Blutung in der Literatur bereits beschrieben; der von uns beschriebene Fall untermauert eben diese Korrelation [2, 3, 5, 6, 8, 9].

## Fazit für die Praxis

- Eine Tako-Tsubo-Kardiomyopathie als Komplikation eines neurogenen Schocks bei schwerem Schädel-Hirn-Trauma oder Hirnstammverletzung sollte bei entsprechendem Traumamechanismus als Ursache einer Reanimationspflichtigkeit in Betracht gezogen werden. Das Erkennen des Schockzustands mit der zügigen Therapie des neurogenen Schocks sowie konsequentes Monitoring der kardialen Funktion mittels Herzechokardiographie und Elektrokardiographie sind hier von großer Wichtigkeit und unterstreichen den Stellenwert der POCUS im Schockraum.
- Es empfiehlt sich, das gesamte Schockraumteam bezüglich des Auftretens, der Pathophysiologie und Therapie des myokardialen Stresszustands bei schwerem Schädel-Hirn-Trauma und neurogenem Schock zu schulen. Eine kontinuierliche kardiovaskuläre Überwachung mit standardmäßiger Durchführung eines Herzechos bei allen Patienten mit Schädel-Hirn-Trauma bei Ankunft im Schockraum sowie Verlaufskontrollen der kardialen Enzyme wären zu empfehlen.

## Supplementary Information

Transthorakale Echokardiographie im Schockraum: Apical ballooning syndrome

## References

[1] Anderson LD, D’alonzo RT (1974) Fractures of the odontoid process of the axis. J Bone Joint Surg Am 56:1663–1674 4434035

[2] Cheah CF, Kofler M, Schiefecker AJ et al (2017) Takotsubo Cardiomyopathy in Traumatic Brain Injury. Neurocrit Care 26:284–291 28000134 10.1007/s12028-016-0334-y PMC5334445

[3] Gopinath R, Ayya SS (2018) Neurogenic stress cardiomyopathy: What do we need to know. Ann Card Anaesth 21:228–234 30052207 10.4103/aca.ACA_176_17 PMC6078016

[4] Krueger A, Frink M, Kiessling A et al (2013) Schockraummanagement. Chirurg 84:437–450 23553150 10.1007/s00104-012-2384-9 PMC7096044

[5] Lyon AR, Bossone E, Schneider B et al (2016) Current state of knowledge on Takotsubo syndrome: a Position Statement from the Taskforce on Takotsubo Syndrome of the Heart Failure Association of the European Society of Cardiology. Eur J Heart Fail 18:8–27 26548803 10.1002/ejhf.424

[6] Núñez-Ramos JA, Aguirre-Acevedo DC, Pana-Toloza MC (2023) Point of care ultrasound impact in acute heart failure hospitalization: A retrospective cohort study. Am J Emerg Med 66:141–145 36753930 10.1016/j.ajem.2023.01.047

[7] Schacher S, Glien P, Kogej M et al (2019) Strukturierte Übergabeprozesse in der Notaufnahme. Notfall Rettungsmed 22:3–8

[8] Wang F, Darby J (2021) Case Report: Takotsubo Cardiomyopathy After Traumatic Brain Injury. Front Neurol 12:727754 34603185 10.3389/fneur.2021.727754 PMC8479872

[9] Krishnamoorthy V, Chaikittisilpa N, Lee J, Mackensen GB, Gibbons EF, Laskowitz D, Hernandez A, Velazquez E, Lele AV, Vavilala MS (2020) Speckle Tracking Analysis of Left Ventricular Systolic Function Following Traumatic Brain Injury: A Pilot Prospective Observational Cohort Study. J Neurosurg Anesthesiol 32:156–161. 10.1097/ana.0000000000000578 30676403 10.1097/ANA.0000000000000578 PMC6646112

